# Influence of demographic and disease parameters on virological response to once- vs twice-daily darunavir/ritonavir (DRV/r) at week 48 in ODIN

**DOI:** 10.1186/1758-2652-13-S4-P25

**Published:** 2010-11-08

**Authors:** M Sension, C Arns Da Cunha, P Domingo, K Supparatpinyo, T Van De Casteele, P De Doncker, F Tomaka

**Affiliations:** 1 Comprehensive Care Center, Fort Lauderdale, FL, USA; 2 Centro Medico Sao Francisco, Curitiba, Brazil; 3 Autonomous University of Barcelona, Barcelona, Spain; 4 Chiang Mai University, Chiang Mai, Thailand; 5 Tibotec BVBA, Beerse, Belgium;; 6 Tibotec Inc., Titusville, NJ, USA

## Background

ODIN was a Phase IIIb, randomised, open-label trial showing non-inferiority of DRV/r 800/100mg qd vs 600/100mg bid (+ ≥2 NRTIs) in treatment-experienced HIV-1-infected adults with no DRV resistance-associated mutations at screening. We evaluated the effect of demographic and baseline (BL) disease parameters on virological response (VR) of qd vs bid DRV/r at Week 48.

## Methods

Week 48 VR (HIV-1 RNA <50 copies/mL; ITT-TLOVR) was analysed by subgroups including gender, age, race, HIV clade, BL HIV-1 RNA and CD4 count.

## Results

Week 48 VR was 72.1% for DRV/r qd vs 70.9% for bid (95% CI = -6.1 to 8.5%). Week 48 VR by gender, age, race, HIV clade, BL CD4 count and HIV-1 RNA is reported (Table 1).

**Table 1:** % patients with HIV-1 RNA <50 copies/mL at Week 48

	DRV/r qd		DRV/r bid		
*Baseline factor*	N	% response	N	% response	Difference in response (95% CI)

HIV-1 RNA (as stratified) (copies/mL)					

≤50,000	222	78.4	224	76.8	1.6 (-6.2, 9.4)

>50,000	72	52.8	72	52.8	0.0 (-16.4, 16.4)

*CD4 count (cells/mm^3^)*					

<50	13	53.8	3	100	-46.2 (-109.6, 17.3)

50-<100	36	58.3	35	57.1	1.2 (-22.2, 24.6)

100-<200	76	77.6	77	67.5	10.1 (-4.1, 24.3)

200-<350	108	72.2	107	74.8	-2.5 (-14.4, 9.3)

≥350	61	77.0	74	74.3	2.7 (-12.0, 17.4)

*Gender*					

Female	115	69.6	98	69.4	0.2 (-12.3, 12.7)

Male	179	73.7	198	71.7	2.0 (-7.0, 11.1)

*Age (years)*					

≤30	35	71.4	35	60.0	11.4 (-11.0, 33.9)

30-≤45	180	75.6	169	72.8	2.8 (-6.4, 12)

45-≤55	64	60.9	72	72.2	-11.3 (-27.2, 4.6)

55-≤65	14	78.6	18	66.7	11.9 (-20.6, 44.4)

>65	1	100	2	100	0 (0, 0)

*Race*					

Black	83	62.7	72	65.3	-2.6 (-17.9, 12.7)

Caucasian	102	71.6	110	69.1	2.5 (-9.9, 14.9)

Hispanic	47	72.3	59	67.8	4.5 (-13.2, 22.3)

Asian	48	89.6	41	87.8	1.8 (-11.6, 15.1)

Other	14	71.4	14	78.6	-7.1 (-40.7, 26.4)

*Clade*					

B	179	70.4	199	64.3	6.4 (-3.4, 15.6)

Non-B	115	74.8	97	84.5	-9.8 (-20.7,1.2)

## Conclusions

VR was comparable with DRV/r qd and bid regardless of subgroup analysed. As the trial was not powered for treatment comparisons in subgroups, and as numbers were small for some subgroups, these exploratory findings should be interpreted with caution.

